# Width Dependent Elastic Properties of Graphene Nanoribbons

**DOI:** 10.3390/ma14175042

**Published:** 2021-09-03

**Authors:** George Kalosakas, Nektarios N. Lathiotakis, Konstantinos Papagelis

**Affiliations:** 1 Materials Science Department, University of Patras, GR-26504 Rio, Greece; 2 Theoretical and Physical Chemistry Institute, National Hellenic Research Foundation, Vass. Constantinou 48, GR-11635 Athens, Greece; lathiot@eie.gr; 3 School of Physics, Department of Solid State Physics, Aristotle University of Thessaloniki, GR-54124 Thessaloniki, Greece; kpapag@physics.auth.gr

**Keywords:** 2D materials, mechanical response, uniaxial tension, numerical simulations

## Abstract

The mechanical response of graphene nanoribbons under uniaxial tension, as well as its dependence on the nanoribbon width, is presented by means of numerical simulations. Both armchair and zigzag edged graphene nanoribbons are considered. We discuss results obtained through two different theoretical approaches, viz. density functional methods and molecular dynamics atomistic simulations using empirical force fields especially designed to describe interactions within graphene sheets. Apart from the stress-strain curves, we calculate several elastic parameters, such as the Young’s modulus, the third-order elastic modulus, the intrinsic strength, the fracture strain, and the Poisson’s ratio versus strain, presenting their variation with the width of the nanoribbon.

## 1. Introduction

Since the isolation of graphene, the first atomically thin two-dimensional material realized experimentally, an enormous number of investigations have explored its fascinating properties. Graphene has exceptional electronic [1,2,3,4,5] and thermal [6,7,8,9] transport characteristics. Besides, its vibrational [10,11,12,13,14], mechanical [15,16,17,18,19,20,21,22,23,24,25,26], and optical [27,28,29,30,31,32,33] properties have been extensively studied both experimentally and theoretically. From the theoretical perspective, several force fields have been designed [20,34,35,36,37,38] that are able to sufficiently describe particular features of this nanomaterial or, more generally, of carbon condensed phases. Various dynamical and structural properties of graphene have been examined [39,40,41,42], as well as the influence of different kinds of defects [43,44,45,46,47,48,49,50,51,52]. Graphene has been used in a number of devices and applications, for example, in integrated circuits [53], sensors/biosensors [54,55,56], detectors [57,58], etc.

Graphene nanoribbons (GNRs) are narrow stripes of graphene having a width in the nanometer scale [59]. There are two main routes of GNR production: a top-down approach using either lithography for etching graphene [60,61] or carbon nanotube unzipping [62,63] and a bottom-up synthesis using appropriate precursor molecules [64,65,66]. The second method provides a controllable fabrication of GNRs with well defined widths and edge structures. Both armchair edged nanoribbons (AGNRs) and zigzag edged nanoribbons (ZGNRs) have been synthesized (see, e.g., Ref. [67] and references therein).

GNRs exhibit a richer behavior than graphene, as their properties can be tuned through engineering of their width and edge structure. For example, due to quantum confinement, narrow graphene nanoribbons present a semiconducting electronic structure with increasing energy band gap as the nanoribbon width decreases [60,61,68]. As a result, structural, vibrational, and electronic properties of GNRs and their applications in devices have been extensively considered [69,70,71,72,73,74,75,76,77].

The mechanical response of graphene nanoribbons under uniaxial tension has also received considerable attention. One of the early studies on this subject presents numerical results on the size dependence of Young’s modulus and Poisson’s ratio for square-shaped GNRs, using both molecular dynamics (MD) simulations with the AIREBO force field and energy calculations through the orthogonal tight-binding method [78]. These different methods showed that the Young’s modulus of GNRs increases with the nanoribbon size, while the Poisson’s ratio decreases, for both armchair and zigzag directions, and the obtained elastic parameters converge to the corresponding bulk values at large widths, above 10 nm [78].

At the same time, another investigation of hydrogen-passivated ZGNRs under relatively small uniaxial stresses, using the PBE functional within the Generalized Gradient Approximation (GGA) in density functional theory (DFT), presented a decrease of both the Young’s modulus and the Poisson’s ratio with the ribbon width, for widths up to 2 nm [79]. In the same theoretical framework of GGA-PBE density functional theory, a study of hydrogen-passivated nanoribbons presents stress-strain data for different ribbon widths, up to about 3 nm for ZGNRs and 1.3 nm for AGNRs, and finds that by increasing width the Young’s modulus decreases slightly for AGNRs but stronger for ZGNRs, while the Poisson’s ratio decreases for AGNRs, but it is insensitive to width for ZGNRs [80].

Results regarding the stress-strain response of AGNRs and ZGNRs of different widths, ranging from 1 to 9 nm, have also been calculated through atomistic molecular mechanics simulations using the REBO force field [81]. The Young’s modulus was found to decrease with the ribbon width for unpassivated ZGNRs and AGNRs, as well as for hydrogen-passivated ZGNRs, while it was increasing for hydrogen-passivated AGNRs. The fracture strain decreased with the width in all cases, though it seems that it was not converging to the bulk values as the width increases, while the intrinsic strength increases with the width except for the case of hydrogen-passivated ZGNRs where it was decreasing [81]. MD simulations using the AIREBO potential in Ref. [82] have shown increase of the Young’s modulus with width for both AGNRs and ZGNRs, while both the fracture strain and the intrinsic strength decay by increasing width for ZGNRs and are almost insensitive on ribbon’s width for AGNRs.

Numerical data from a structural mechanics approach examining GNRs of different lengths and widths up to 10 nm has found that the Young’s modulus increases with the width in zigzag edged nanoribbons, while it exhibits a non-monotonous behavior for relatively narrow AGNRs [83]. Finally, an atomic scale finite element method has calculated stress-strain curves for ribbons of different widths and found a response insensitive to width for AGNRs, while the Young’s modulus and the intrinsic strength of ZGNRs is a decreasing function of ribbon width, in contrast to the width independent case of AGNRs [84].

Further, the effects of various defects, such as Stone-Wales defects [85], large vacancy rings and different kinds of N doping [86], edge defects [80], and defective GNRs with coved edges [87], on the mechanical behavior of GNRs under uniaxial tension have been examined. The response of uniaxially compressed graphene nanoribbons has been also investigated [52,88].

Here, we present extensive numerical computations of the mechanical behavior of ZGNRs and AGNRs when a tensile load is applied uniaxially on their ends, and we determine the corresponding elastic constants and their dependence on ribbon width. As already mentioned, both the edge structure and the nanoribbon width provide controllable parameters that affect materials’ properties. We apply first principle methods as well as atomistic MD simulations using appropriate potentials for describing bond stretchings and angle bendings within the graphene plane. In the following section, we outline the computational methods used in this work, and the next section contains a discussion of our results. The final section concludes our study.

## 2. Methods

### 2.1. Density Functional Theory Calculations

The strain response of ZGNRs and AGNRs that are not passivated was studied by DFT theoretical simulations at the GGA level and the PBE functional [89]. We employed the Quantum-Espresso computer code [90] with an ultra-soft RRKJ-type pseudopotential [91]. This combination has been proven to reproduce accurately structural, mechanical, vibrational, and thermodynamic properties of carbon allotropes [49,92].

**Figure 1:**
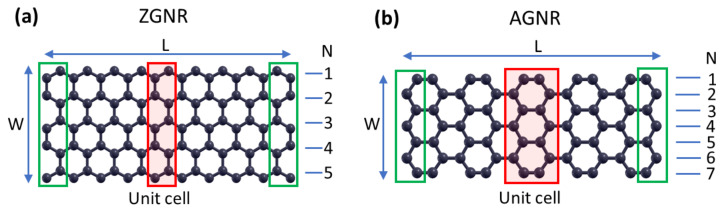
Examples of: (**a**) a zigzag nanoribbon and (**b**) an armchair nanoribbon. L is the length of the nanoribbon, and W is its width. The unit-cell employed in DFT calculations is shown with red boxes. The green boxes contain the terminal atoms on which forces were applied in the atomistic MD simulations. The convention of layer numbering (N) is also shown.

In Figure 1, we show examples of the employed unit-cells (see red boxes) for the cases of ZGNRs and AGNRs. Graphene nanoribbons are 1D periodic systems and, as can be seen from Figure 1, the dimension of the unit-cell along the direction of periodicity for the AGNRs is roughly two times larger than that of ZGNRs (4.27 Å and 2.46 Å respectively). Accordingly, we used 8 k-points for the AGNRs and 16 for the ZGNRs. Concerning the vertical to the nanoribbon direction, in order to isolate the nanoribbon, a periodicity was assumed with an empty space of at least 15 Å between a nanoribbon and its images due to periodicity.

Initially, structures were optimized as far as the atomic positions and the unit-cell are concerned. Subsequently, strain was applied along the direction of periodicity by increasing the unit-cell dimension and keeping it frozen in the calculations while all atomic positions were allowed to relax. This procedure allows for the width of the nanoribbon to adjust. We performed calculations for nanoribbons of several widths, extending up to more than 5 nm for ZGNRs and up to more than 2 nm for AGNRs, and for strains in the range 0–30%.

For all different widths, the dependence of the total energy on strain was obtained. Typically, this dependence is parabolic for small strains, and, as strain increases, the curve bends and reaches a maximum value. We assume that this maximum corresponds to fracture strain. However, we should keep in mind that the imposed periodicity restricts the fracture mechanism, since it does not allow fracture paths that alter this periodicity. Thus, one expects that the so-obtained fracture strains might be, in some degree, overestimated. Having the total energy for several values of strain, the force acting to the vertical to the strain unit-cell edges was obtained numerically as the first derivative of the total energy with respect to the unit-cell dimension along the strain direction. Then, the two-dimensional (2D) stress was calculated by dividing this force with the vertical to the strain unit-cell dimension (i.e., the width of the nanoribbon).

### 2.2. Molecular Dynamics Simulations

For the MD simulations, we used the empirical force fields introduced in Ref. [20] for the description of in-plane motion in graphene. These potentials concern the bond stretching and valence angle bending of planar sp${}^{2}$ carbons within a graphene sheet, and they have been derived through fitting with numerical data obtained from first principles’ methods.

Using the layer numbering depicted in Figure 1, we have considered ZGNRs with sizes 87 × N, where N = 4, 6, 8, 10, 12, 20, 40, 60, and 80 (see Figure 1a), and AGNRs with sizes 86 × N, where N = 5, 7, 9, 11, 13, 21, 41, 61, and 81 (see Figure 1b), in order to examine nanoribbons of varying widths.

The response of GNRs under uniaxial tension is calculated by applying constant forces at all atoms on the ends vertical to the long direction, of length L, of the nanoribbon (shown with green boxes in Figure 1). Opposite forces are applied at the different ends, pulling the corresponding atoms away. All other atoms of the GNR, including those on the long edges, are free of any force or other constrains, just interacting through the considered force fields with their neighboring atoms of the structure. Applying a friction term at each atom of GNR, the evolution of the system is followed numerically through Newton’s equations of motion, until reaching the equilibrium corresponding to the applied tensile forces [20]. The used dissipation coefficient was 10 ps${}^{-1}$, but we note that this choice has not any effect on the equilibrium state of the system, only affecting the transient behavior and the time to reach equilibrium. Then, the average strain along the long direction of the nanoribbon is calculated at this equilibrium state, while the corresponding 2D stress is obtained by the total force applied at each end (i.e., the sum of the forces acting at all atoms of the GNR’s end) divided by the original nanoribbon width W (the width before the deformation, as usually considered when calculating the nominal stress).

## 3. Results and Discussion

### 3.1. Stress-Strain Curves

The mechanical response of graphene nanoribbons is quantified through the corresponding stress-strain curves. The calculated response under uniaxial tension is presented in this subsection. A number of elastic parameters, such as the Young’s modulus, the third-order elastic modulus, the intrinsic strength, and the fracture strain, are obtained through the stress-strain curves and will be discussed in the following subsections.

**Figure 2:**
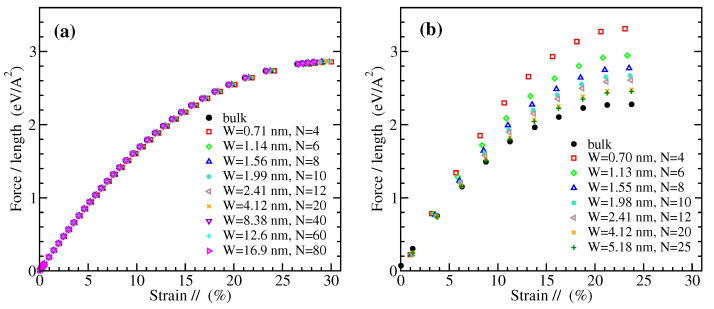
The 2D stress (force per unit length) as a function of strain parallel to the loading direction for ZGNRs of different widths W, obtained by (**a**) MD simulations and (**b**) DFT calculations. The layer numbering N is shown in Figure 1a.

**Figure 3:**
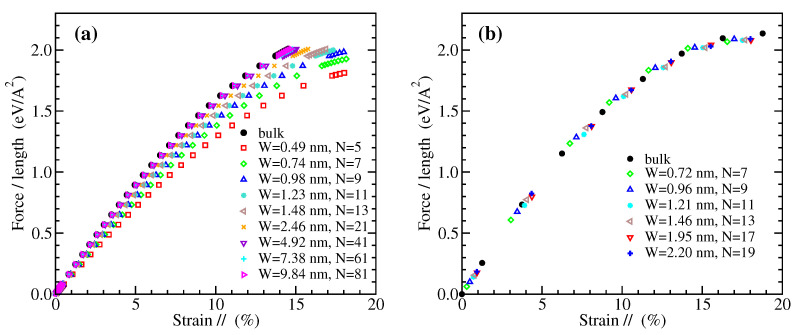
The 2D stress (force per unit length) as a function of strain parallel to the loading direction for AGNRs of different widths W, obtained by (**a**) MD simulations and (**b**) DFT calculations. The layer numbering N is shown in Figure 1b.

The nominal 2D stress-strain curves for zigzag graphene nanoribbons of various widths are shown in Figure 2 and for armchair nanoribbons in Figure 3. The left panels in these figures present results obtained from MD simulations, while the right panels from DFT computations, as described in the previous section. The widths of the examined nanoribbons range from the subnanometer scale up to around 17 nm (10 nm) for ZGNRs (for AGNRs) in the MD case or a few nm in the DFT case. The GNRs examined using DFT here are almost twice as wide as those considered in earlier studies [79,80]. In all cases, the results for the wider GNRs considered in this work converge to the corresponding bulk data [20], which are explicitly shown here (see the black filled circles in Figure 2 and Figure 3) for comparison.

The results of Figure 2 demonstrate that the mechanical response of ZGNRs appears to be rather insensitive to the ribbon width W in the MD simulations, while there is a smooth dependence in the DFT calculations. The other way around is for the AGNRs, as can be seen from Figure 3. Our MD derived stress-strain response of ZGNRs and AGNRs is in qualitative agreement with molecular mechanics calculations that employ energy minimization using the REBO potential, as shown in Figure 4 of Ref. [81]. On the other hand, similar trends with our DFT data have been observed in Figure 4 of Ref. [80], apart from the fact that a small dependence of the mechanical response of AGNRs on their width W has been observed there. However, we note that hydrogen-passivated GNRs have been considered in the DFT results discussed in that work. It is worth mentioning that an insensitivity of the mechanical behavior of AGNRs on their width has been also found using an atomic scale finite element method, while a qualitatively similar picture with our DFT data has been observed in the case of ZGNRs, though with a weaker width dependence, as can be seen from Figure 8 of Ref. [84].

### 3.2. Young’s Modulus

**Figure 4:**
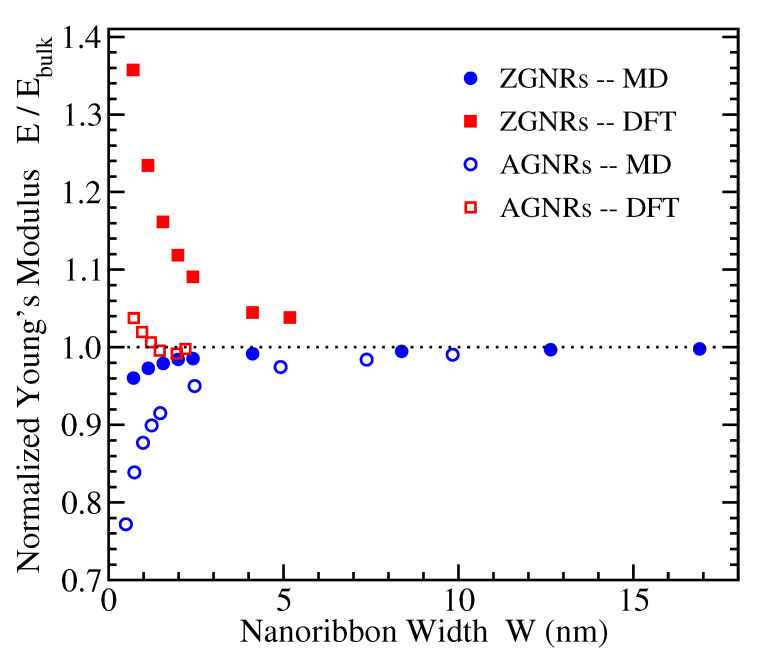
The ratio $E/E_{\mathrm{bulk}}$ of the 2D Young’s moduli of nanoribbons, E, and bulk graphene, $E_{\mathrm{bulk}}$, obtained from the MD (circles) and DFT (squares) simulations, for ZGNRs (filled symbols) and AGNRs (open symbols), as a function of nanoribbon width.

The slope of the stress-strain curves at small strains provides the Young’s modulus *E* of a material. The 2D Young’s moduli of both armchair and zigzag edged GNRs have been calculated from the data presented in the previous subsection, and their dependence on nanoribbon width is depicted in Figure 4. In that figure, the values of *E* have been normalized to the 2D Young’s modulus of bulk graphene, which has been calculated to be $E_{bulk}=320$ N/m within both DFT and MD approaches considered here [20]. This value is within the experimental estimate [17] of 340 ± 50 N/m, corresponding to a 3-dimensional Young’s modulus of 1.0 ± 0.1 TPa if one considers the thickness of single layer graphene to be 3.35 Å.

Figure 4 shows that, as the GNR width increases, the normalized Young’s modulus tends to 1 in all cases, as expected. However, the DFT and MD results demonstrate opposite trends. In particular, the DFT data reveal an increasing Young’s modulus as the width of the GNR decreases, which is more evident for ZGNRs (reaching more than 30% increase with respect to the bulk value for the smallest ribbon considered here), while the MD computations result in decreasing values of *E* as W decreases, which is more evident in the case of AGNRs (showing more than 20% decrease from the bulk value for the smallest nanoribbon examined).

This contradicting qualitative behavior is in accordance to the observed dependence of *E* in previous investigations. Other DFT calculations in hydrogen-passivated nanoribbons have also found larger values of *E* for smaller ribbon widths for the case of ZGNRs (see Figure 3 of Ref. [79]), while the width dependence of both ZGNRs and AGNRs shown in Figure 5 of Ref. [80] exhibits a similar behavior as that presented by our DFT data in Figure 4. On the contrary, MD simulations using the AIREBO potential resulted in increasing Young’s modulus with W for both AGNRs and ZGNRs (see Figure 10 of Ref. [82]), as also shown in our MD data. Another study has also shown increased values of *E* with the ribbon size but for square-shaped AGNRs and ZGNRs using both MD calculations with the AIREBO potential and tight-binding energy computations, as well [78].

Interestingly, the molecular mechanics results of Ref. [81], demonstrating qualitatively similar stress-strain curves as our MD findings, lead to decreasing Young’s modulus with W for unpassivated ZGNRs and AGNRs. Decaying values of *E* by increasing W have been also found for ZGNRs in the atomic scale finite element method of Ref. [84], while there is no sensitivity of *E* to W in the AGNRs’ case. However, the obtained values of $E_{bulk}$ in the last two works are well below the experimentally determined [17] and widely accepted Young’s modulus of bulk graphene mentioned above.

### 3.3. Third-Order Elastic Modulus

The stress-strain response of GNRs can be accurately described by the following quadratic relation:

(1) $$\sigma =E\cdot \varepsilon +D\cdot \varepsilon^{2},$$

where σ corresponds to the 2D stress, ε is the strain (expressed in pure values, not in percentages as in Figure 2 and Figure 3, for example a value of 0.1 should be used instead of 10%), *E* is the 2D Young’s modulus, and *D* is the 2-dimensional third-order elastic modulus describing the departure of the mechanical response from the linear behavior.

Through fitting of the stress-strain curves presented in Figure 2 and Figure 3 with Equation (1), the respecting values of D have been derived for all GNRs examined here. In this fitting procedure, just one fitting parameter has been considered, the elastic constant *D*, since the values of *E* have been independently determined through the stress-strain data at small strains as discussed in the previous subsection. We note that the third-order elastic modulus is negative for graphene, as well as for GNRs.

**Figure 5:**
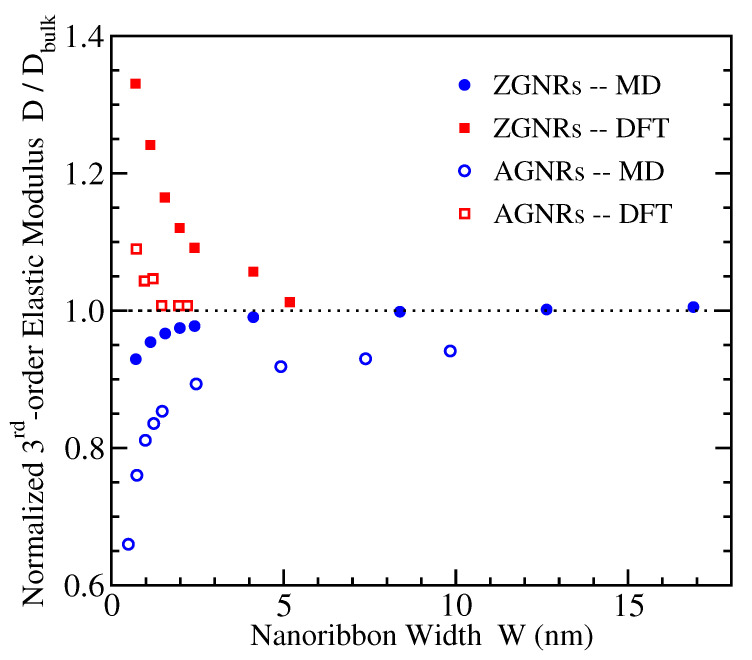
The ratio $D/D_{\mathrm{bulk}}$ of the 2-dimensional third order elastic moduli of nanoribbons, D, and bulk graphene, $D_{\mathrm{bulk}}$, obtained from the MD (circles) and DFT (squares) simulations, for ZGNRs (filled symbols) and AGNRs (open symbols), as a function of nanoribbon width.

Figure 5 depicts the normalized values of *D*, divided over the corresponding quantities $D_{bulk}$ of bulk graphene, which provide their large width limitings values. These bulk values have been calculated −700 N/m for AGNRs and −670 N/m for ZGNRs using DFT, and −670 N/m for AGNRs and −560 N/m for ZGNRs using MD [20]. An available experimental estimate of the 2D value of third-order elastic modulus is −690 N/m [17].

Once more, we see that the calculated third-order elastic moduli converge to the corresponding bulk quantities when the width *W* increases, as the normalized values tend to 1. However, the DFT and MD results show again opposite trends, in a similar way as discussed for the Young’s moduli in the previous subsection. Existing investigations of the mechanical properties of graphene nanoribbons [78,79,80,81,82,83,84] have not reported results for the third-order elastic modulus and its dependence on width in order to compare our findings with other available data.

### 3.4. Fracture Strain

The fracture strain is the maximum strain that can be sustained by a GNR before failure. It is given by the abscissa of the last point of the stress-strain curve, immediately before failure. In the atomistic MD simulations, which are less computer-time consuming than the corresponding DFT calculations, there is an increased density of points near fracture, as can be clearly seen for example in Figure 3a, in order to locate more precisely the fracture point.

**Figure 6:**
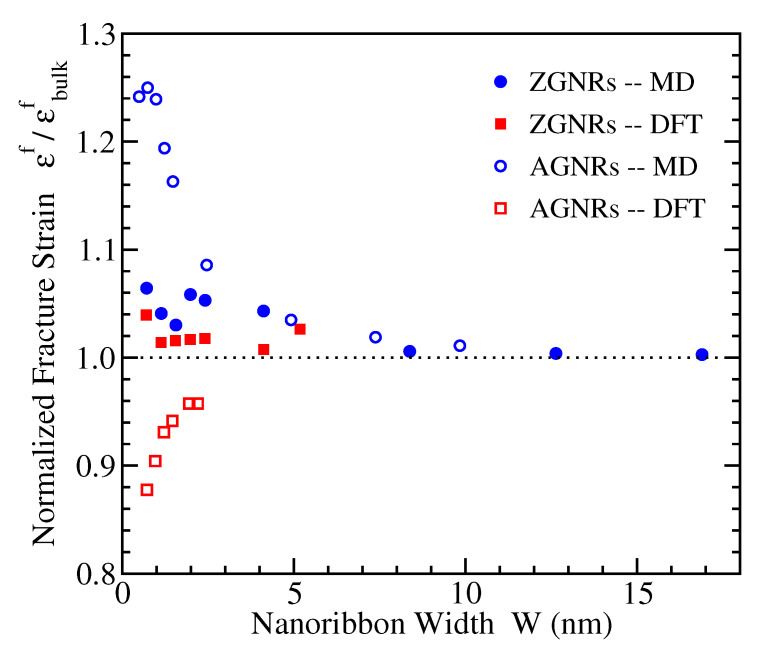
The ratio $\varepsilon^{f}/\varepsilon_{\mathrm{bulk}}^{f}$ of the fracture strains of nanoribbons, $\varepsilon^{f}$, and bulk graphene, $\varepsilon_{\mathrm{bulk}}^{f}$, obtained from the MD (circles) and DFT (squares) simulations, for ZGNRs (filled symbols) and AGNRs (open symbols), as a function of nanoribbon width.

Figure 6 depicts the fracture strains $\varepsilon^{f}$ for the two families of GNRs with the different structural edges, normalized to the corresponding bulk values $\varepsilon_{\mathrm{bulk}}^{f}$, which in the considered MD (DFT) approach have been found [20] to be more than 28% (around 24%) and around 14.5% (around 19%) for ZGNRs and AGNRs, respectively. In this case, the DFT and MD results agree that narrow zigzag nanoribbons have fracture strains slightly above the corresponding bulk values and are weakly dependent on width. Regarding the armchair nanoribbons, which show stronger width dependence, the two methods give opposite trends; $\varepsilon^{f}$ decreases (increases) for smaller widths in DFT (MD) up to more than 10% (more than 20%) of the corresponding bulk value.

We are not aware of other DFT results regarding the fracture strain or the intrinsic strength (see next subsection) of GNRs. Molecular dynamics simulations with the AIREBO force field have found that, for AGNRs, there is no dependence of $\varepsilon^{f}$ on the width, while, for ZGNRs, the fracture strain decreases with W reaching values of around 16% for a 12 nm wide zigzag nanoribbon (see Figure 12 of Ref. [82]). However, such a value of fracture strain is considerably below the calculated by different methods $\varepsilon_{\mathrm{bulk}}^{f}$ of graphene under tensile load in this direction, which is well above 20% [16,19,20,80]. Atomistic molecular mechanics calculations using the REBO potential have found for unpassivated ZGNRs with widths up to 8 nm a qualitatively similar behavior as in our MD data (see Figure 7 of Ref. [81]), while, for unpassivated AGNRs the fracture strain shows a slight decrease on W, but the presented results do not seem to converge to the corresponding bulk values.

### 3.5. Intrinsic Strength

**Figure 7:**
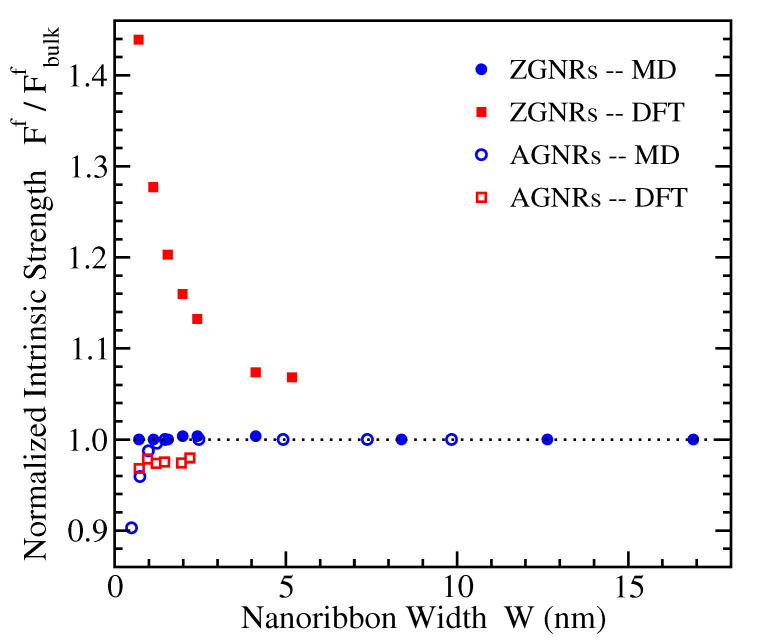
The ratio $F^{f}/F_{\mathrm{bulk}}^{f}$ of the fracture stresses of nanoribbons, $F^{f}$, and bulk graphene, $F_{\mathrm{bulk}}^{f}$, obtained from the MD (circles) and DFT (squares) simulations, for ZGNRs (filled symbols) and AGNRs (open symbols), as a function of nanoribbon width.

The intrinsic strength, also referred to as fracture stress or tensile strength, represents the maximum stress that a material can withstand, while for larger stresses it fails. It is obtained by the stress-strain curve through the last point’s, just before failure, ordinate. The DFT and MD derived values of intrinsic strengths for ZGNRs and AGNRs and their variation with the nanoribbon width are shown in Figure 7, once more normalized to the respective bulk values. The bulk graphene values for uniaxial tension along the same direction as in the AGNRs are around 34 N/m using DFT and around 32 N/m using MD, while for the perpendicular direction, i.e. the same direction as in ZGNRs, are 38 N/m and 45 N/m, respectively [20]. We mention here that the experimentally estimated intrinsic strength of graphene is 42 ± 4 N/m, corresponding to an effective 3-dimensional value of 130 ± 20 GPa [17].

The numerical results presented in Figure 7 show that, for AGNRs, the fracture stresses show almost no dependence on the ribbon width in DFT, while, in MD, there is a small variation only for extremely narrow widths, in the subnanometer scale. For ZGNRs the MD intrinsic strengths seem to be insensitive to the width, but the DFT data show a strong increase of the fracture stress by reducing W, that reach more than 40% increase with respect to the bulk value for the narrower nanoribbons considered here.

In qualitative agreement to our DFT data are results from MD simulations (see Figure 11 of Ref. [82]) and from finite element approaches (see Figure 9, right panel of Ref. [84]) showing no dependence of AGNRs’ intrinsic strength on W and decreasing values of $F^{f}$ with ribbon width for ZGNRs. Contrary to these results, molecular mechanics calculations for unpassivated GNRs have found increasing fracture stresses with W, an effect which is stronger for AGNRs and very small for ZGNRs (see Figure 9 of Ref. [81]).

### 3.6. Poisson’s Ratio

The Poisson’s ratio, ν, represents the relative contraction in the direction perpendicularly to the applied load over the relative extension (strain) in the direction of the tensile load. It is given by the quantity $\nu =-\varepsilon_{⊥}/\varepsilon_{//}$, where the $\varepsilon_{⊥}$ is negative due to the lateral contraction. Within our DFT and MD approaches, we have calculated the lateral strains and the corresponding Poisson’s ratios for the examined GNRs under uniaxial tension. In the MD case, the lateral strain at the middle of the length of the nanoribbon is computed in order to avoid end effects. Though the Poisson’s ratio is usually referred to small deformations, here we present results for the whole range of strains up to materials’ failure, since ν exhibits a strong dependence on strain.

**Figure 8:**
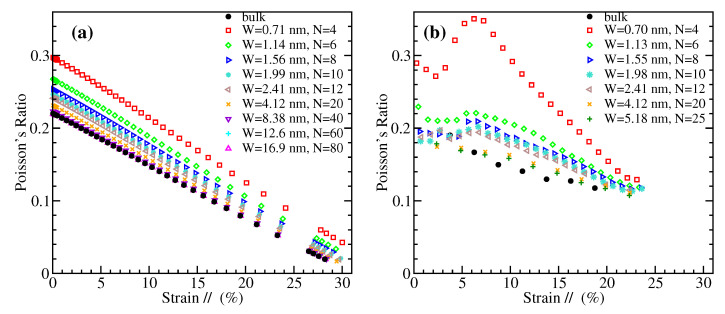
Poisson’s ratio of ZGNRs of different widths as a function of strain parallel to the loading direction, obtained by (**a**) MD simulations and (**b**) DFT calculations.

**Figure 9:**
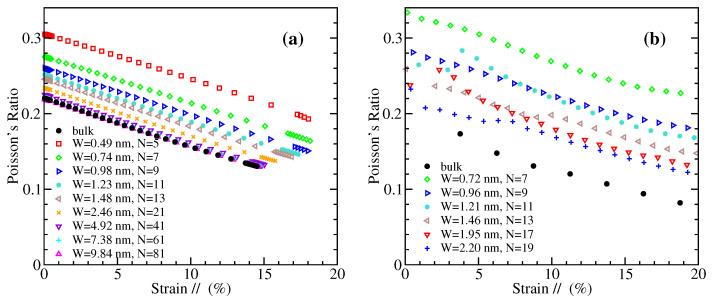
Poisson’s ratio of AGNRs of different widths as a function of strain parallel to the loading direction, obtained by (**a**) MD simulations and (**b**) DFT calculations.

Figure 8 and Figure 9 depict the variation of the Poisson’s ratio with strain for zigzag and armchair nanoribbons, respectively. The results obtained by MD are shown in the left panels, and those from DFT in the right ones. Both methods demonstrate that in general narrower GNRs exhibit larger Poisson’s ratios, and there is a decrease of ν with increasing width towards the bulk values which are shown by black filled circles in these figures. The MD data show a smooth, almost linear, dependence of Poisson’s ratio on strain, for both ZGNRs and AGNRs. A roughly similar behavior is shown by DFT in the case of AGNRs, but with larger Poisson’s ratios than the corresponding MD values for the narrower nanoribbons (Figure 9). For ZGNRs, the DFT results give a more irregular dependence on strain, which is more evident for the smallest width examined (Figure 8), perhaps due to the unpassivated edges of the considered nanoribbons.

A decrease of the Poisson’s ratio with the width has been also found in Ref. [79] for ZGNRs using DFT, but this behavior depends on the width definition (see Figure 4 of that work). Another DFT study shows a decrease of ν with *W* for AGNRs, but almost no dependence for ZGNRs, while in both cases the Poisson’s ratio decays with strain (see Figure 6 of Ref. [80]). We mention that hydrogen-passivated GNRs have been considered in these two works. For AGNRs, the strain dependence of ν in Ref. [80] is qualitatively similar to our DFT data of Figure 9b, but this is not the case for the ZGNRs. Perhaps the passivation/unpassivation has stronger effects on ν for narrow zigzag nanoribbons. A decrease of the Poisson’s ratio with the size of GNR has been also observed in the atomistic simulations of Ref. [78], but in square-shaped ribbons.

**Figure 10:**
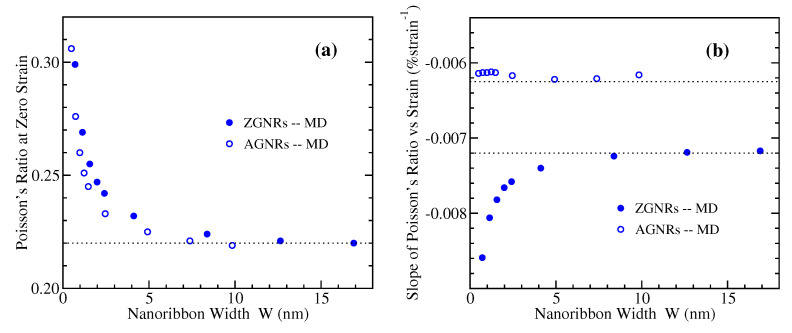
Dependence on width of (**a**) the intercept and (**b**) the slope, concerning the linear variation of Poisson’s ratio with strain, Equation (2), as obtained by MD simulations for the zigzag (filled circles) and armchair (open circles) GNRs. The horizontal dotted lines denote the corresponding bulk values, which coincide for ZGNRs and AGNRs regarding the intercept, but they differ for the slope.

To further quantify the variation of the Poisson’s ratio with strain for the GNRs of different width, we have fitted by straight lines the MD derived numerical data shown in Figure 8a and Figure 9a. These data are described very well by a linear relation of the form

(2) $$\nu =a\cdot \varepsilon +\nu_{0},$$

where *a* is the slope, and $\nu_{0}$ is the intercept representing the Poisson’s ratio at the zero strain limit. The results of the fitting are shown in Figure 10, where the dependence of the intercept $\nu_{0}$ and the slope *a* on ribbon width is presented. The large width limiting values of bulk graphene are $\nu_{0}=022$ for both armchair and zigzag nanoribbons, while a=−0.0072/%strain for ZGNRs and a=−0.00625/%strain for AGNRs [20]. Figure 10a depicts the increase of zero-strain Poisson’s ratio as the width of GNRs decreases, from the bulk value of 0.22 to around 0.30. Regarding the dependence of the slope *a*, as can be seen from Figure 10b, there is almost no variation with *W* for AGNRs, while the negative slope is steeper for narrower ribbons in the case of ZGNRs.

## 4. Conclusions

We have presented the stress-strain response of graphene nanoribbons of varying widths, theoretically calculated using density functional theory, as well as atomistic molecular dynamics simulations. Results for zigzag and armchair edged nanoribbons have been obtained, and the width dependence of a number of elastic properties has been discussed. The widths of the examined nanoribbons range from tenths of nm up to a few nm in DFT and 10–17 nm in MD, depending on the edge structure. The DFT investigated nanoribbons are almost twice as wide as those considered in similar studies.

All the results presented here smoothly converge to the corresponding bulk data as the width of the nanoribbons increases. The calculated elastic constants of nanoribbons include the Young’s modulus, where a relatively large number of results obtained by various numerical methods are available, as well as the intrinsic strength and fracture strain, where DFT data are lacking, the Poisson’s ratio, where results from atomistic simulations or for unpassivated nanoribbons generally are lacking, and the third-order elastic modulus, which is not much investigated in the literature.

DFT (MD) calculations show stronger dependence on width of the stress-strain curves for zigzag (armchair) nanoribbons and almost width insensitive response for armchair (zigzag) nanoribbons. In general, opposite trends are revealed by the DFT and MD outcomes in many cases. However, this is not so surprising, as for example in the case of Young’s modulus, which is well investigated, this is in accordance with existing data.

## Data Availability

The data of this study are available from the corresponding author upon reasonable request.

## Abbreviations

The following abbreviations are used in this manuscript:

GNR	Graphene NanoRibbon
AGNR	Armchair Graphene NanoRibbon
ZGNR	Zigzag Graphene NanoRibbon
MD	Molecular Dynamics
GGA	Generalized Gradient Approximation
DFT	Density Functional Theory
2D	2-Dimensional
